# Relationship between Height-Weight Difference Index and Body-Fat Percentage Estimated by Bioelectrical Impedance Analysis in Thai Adults

**DOI:** 10.1155/2017/7258607

**Published:** 2017-06-11

**Authors:** Kanokkarn Juntaping, Kaweesak Chittawatanarat, Sukon Prasitwattanaseree, Jeerayut Chaijaruwanich, Patrinee Traisathit

**Affiliations:** ^1^Bioinformatics Research Laboratory, Faculty of Science, Chiang Mai University, Chiang Mai, Thailand; ^2^Department of Surgery, Faculty of Medicine, Chiang Mai University, Chiang Mai, Thailand; ^3^Department of Statistics, Faculty of Science, Chiang Mai University, Chiang Mai, Thailand; ^4^Department of Computer Science, Faculty of Science, Chiang Mai University, Chiang Mai, Thailand

## Abstract

**Introduction:**

The height-weight difference index (HWDI) is a new indicator for evaluating obesity status. While body-fat percentage (BF%) is considered to be the most accurate obesity evaluation tool, it is a more expensive method and more difficult to measure than the others.

**Objective:**

Our objectives were to find the relationship between HWDI and BF% and to find a BF% prediction model from HWDI in relation to age and gender.

**Method:**

Bioelectrical impedance analysis was used to measure BF% in 2,771 healthy adult Thais. HWDI was calculated as the difference between height and weight. Pearson's correlation coefficient was used to assess the relationship between HWDI and BF%. Multiple linear and nonlinear regression analysis were used to construct the BF% prediction model.

**Results:**

HWDI and BF% were found to be inverse which related to a tendency toward a linear relationship. Results of a multivariate linear regression analysis, which included HWDI and age as variables in the model, predicted BF% to be 34.508 − 0.159 (HWDI) + 0.161 (age) for men and 53.35 − 0.265 (HWDI) + 0.132 (age) for women.

**Conclusions:**

The prediction model provides an easy-to-use obesity evaluation tool that should help awareness of underweight and obesity conditions.

## 1. Introduction

Obesity is a common problem in many countries and has increasingly become a global epidemic resulting in lower quality of life all over the world. In 2014, the World Health Organization (WHO) reported that about 13%, or one in 10, of the world population aged over 18 (11% men and 15% women) suffered from obesity [1]. This problem is responsible for an increase in the mortality rate from chronic disease (44% from diabetes, 23% from heart disease, and 7% from cancer) [2, 3]. In the Asian community, Thailand ranks second highest behind Malaysia for the number of obese people. The main concern is the apparent increase in the number of children with obesity. A survey in the year 2010 reported 1 in 10 children aged between 1 and 14 in Thailand suffered from obesity [4].

Currently, there are several widely used methods to assess overweightness and obesity in adults. Body-fat percentage (BF%) is an accurate and reliable measurement method but is relatively expensive and difficult to use [5–9]. Dual-energy X-ray absorptiometry (DEXA) is considered as one of the most accurate methods and the gold standard in the measurement of BF%. However, this method is too expensive for regular use, particularly in a resource limited country such as Thailand, whereas bioelectrical impedance analysis (BIA) is less expensive and more practical and has been shown to be moderately accurate in comparison to the gold standard method [10, 11].

The body mass index (BMI) is the most common index for assessing weight status and is calculated from weight (kg) and height (m^2^) [12]. However, this method requires additional devices for measurement. Moreover, BMI cannot be used to distinguish between an obese or overweight individual when a group consists of a population with normal bodyweight but high BF%. This may result in an underestimation of the number of individuals in a population with obesity [13]. To overcome this limitation, the Research Institute for Health Sciences in Thailand came up with a simple index for screening overweightness and obesity called the height-weight difference index (HWDI) by assessing the difference between height (cm) and weight (kg). They also found that HWDI was associated with determining obesity prevalence in ages over 18 [14].

Although there have been previous studies that have analyzed the relationship between BMI and BF% [13, 15, 16], none were found to have focused on the relationship between HWDI and BF%. Our objectives were to find the relationship between HWDI and BF% and to find a BF% prediction model for obesity evaluation.

## 2. Materials and Methods

### 2.1. Study Population

Between 2010 and 2011 at the Faculty of Medicine, Chiang Mai University, adult Thai volunteers were requested via public information posters and the hospital website. A cross-sectional analysis was performed on 2,771 healthy respondents comprising 64% women with a median age of 52 years (interquartile range (IQR) 43–60) and 36% men with a median age of 60 years (IQR 47–68). Volunteers aged less than 18 years or pregnant women were excluded.

### 2.2. Data Measurement

Body weight was measured using the same digital weighing apparatus each time (TCA-200 A-RT; Zepper, Bangkok, Thailand) and recorded in kilograms to one decimal point. Height was measured using a standard stadiometer; the subjects' body positions ensured their head, shoulder blades, buttocks, and heels were touching the board during measurement, recorded in centimeters. HWDI was calculated as the difference between height (cm) and weight (kg) [14].

We used BIA method to estimate BF%. The measurement of bioelectrical impedance depends on the difference in electrical conductivity between fat-free mass and fat, and the technique measures the impedance of an electrical current passed between two electrodes (typically 800 *μ*A; 50 kHz). For single frequency BIA, two electrodes are generally located on the right ankle and the right wrist of an individual. The impedance is related to the volume of a conductor (the human body) and the square of the length of the conductor, a distance which is a function of the height of the subject. BIA analysis most closely estimates body water, from which fat-free mass is then estimated, on the assumption that the latter contains about 73% water. Before analysis, all participants were asked to observe the following pretest guidelines: (1) no prior alcohol consumption within 24 hours; (2) no exercise, caffeine, or food within four hours prior to taking the test; and (3) drinking two to four glasses of water two hours before testing. During the examination, two pairs of sensor electrocardiograph pads were placed on the participants, one on the right wrist and hand and the other on the right foot and ankle; it was necessary for at least 75% of the electrode to be in contact with the participant's skin [17]. BIA offers a reliable option for measuring BF%, and a strong association has been found to exist between BF% and BMI for Thais [18].

### 2.3. Statistical Analysis

All of the continuous variable data were reported as medians and IQRs, and the categorical data were reported as numbers and percentages. The Wilcoxon rank-sum test was used to compare differences between characteristics and gender. Pearson's correlation coefficients (*r*) were calculated to assess the degree of the association between HWDI and BF% in relation to age and gender. Age was divided into three groups (18–39 years, 40–60 years, and over 60 years).

We used regression analysis to examine the relationship between HWDI and BF% performed on men and women separately. Multiple linear regression analysis was first used, followed by an examination of the possibility of a nonlinear relationship existing by including quadratic and cubic forms. Adjusted *R*^2^ and standard error of estimate (SEE) values were used to compare the performance of the predictive model of BF%.

All reports of *p* were two-sided and *p* less than 0.05 was considered statistically significant. All analyses were performed using STATA software version 12.0 (STATA Corp, College Station, Texas, USA) and SPSS version 17.0 (SPSS Inc., Chicago, USA).

## 3. Results

### 3.1. Study Population and Baseline Characteristics

All 2,771 participants in this study were over 18 years and comprised 64% women with a median age of 52 years (IQR, 43–60) and 36% men with a median age of 60 years (IQR, 47–68). The BF% in men was statistically significantly lower than in women (27% and 34%, resp.; *p* < 0.001). The difference in HWDI between men and women was also statistically significant (101 [IQR, 95–107] for men and 98 [IQR, 92–104] for women; *p* < 0.001) (see Table 1).

**Figure 1:**
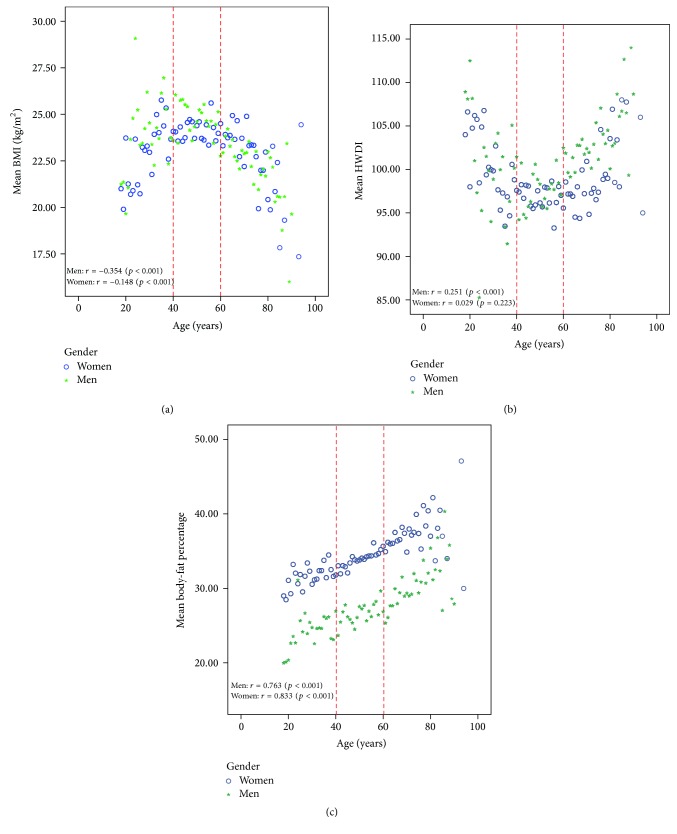
Relationship between (a) mean BMI and age, (b) mean HWDI and age, and (c) mean body-fat percentage and age, stratified by gender, (○) for women and (∗) for men.

Figure 1 shows the relationship between average BMI and age. It was found that, from the age of 18 to 39 years, the mean BMI increases as age increases but, after reaching 60 years of age, the mean BMI decreases as age increases (see Figure 1(a)). The reverse can be found for the relationship between mean HWDI and age.

### 3.2. Relationship between HWDI and BF%

**Figure 2:**
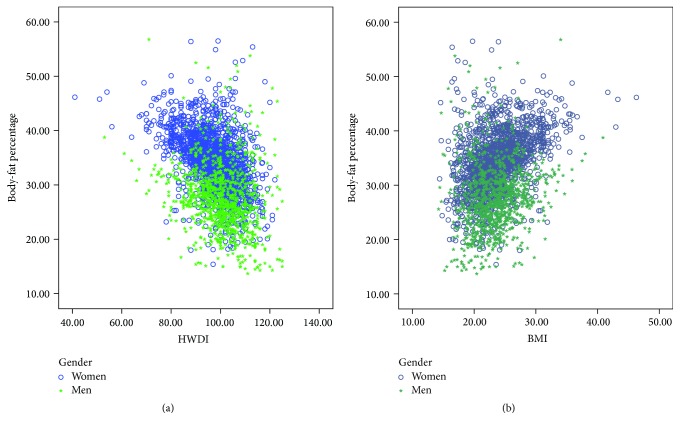
Relationship between (a) HWDI and body-fat percentage and (b) BMI and body-fat percentage, (○) for women and (∗) for men.

**Figure 3:**
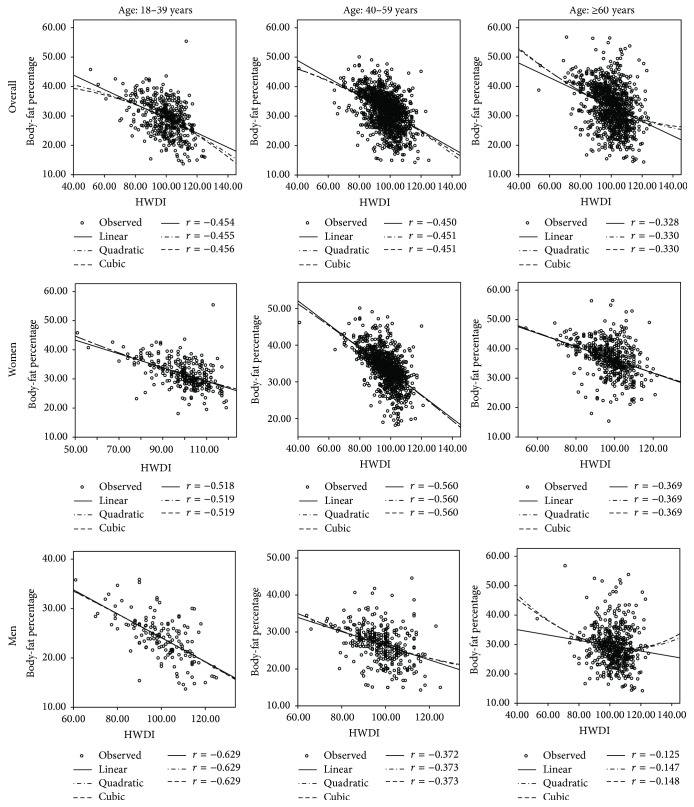
HWDI and body-fat percentage in relation to age and gender.

Figure 2 shows the relationship between HWDI and BF%. Statistically, an inverse relationship between HWDI and BF% was found as HWDI increased while BF% value significantly decreased. Pearson's correlation coefficient (*r*) = −0.200 (*p* < 0.001) in men and *r* = −0.473  (*p* < 0.001) in women. In contrast, the direct relationship was found between BMI and BF%, *r* = 0.144  (*p* < 0.001) in men and *r* = 0.421  (*p* < 0.001) in women. Furthermore, the relationship between HWDI and BF% was statistically significant even when analyzed with respect to age group and gender (*p* < 0.001) (see Figure 3).

### 3.3. The Effect of Age, Gender, and HWDI on BF%

The study of the effect of age, gender, and HWDI on BF% showed all three variables' relationship with BF% to be statistically significant for building a prediction model (*p* < 0.001 for all variables). HWDI and BF% by age and gender resulted in *r* = −0.629/−0.518 (men/women) for the 18–39-year age group, *r* = −0.372/−0.560 for the 40–59-year age group, and *r* = −0.125/−0.369 for age group over 60 years (see Figure 3).

### 3.4. Predictive Modeling of BF% by Gender

In this study, several forms of relationship between HWDI and BF% were studied: linear, quadratic, and cubic. However, Figure 3 shows that the relationship tended to be in linear form more than the others, and so we elected to use a linear form in the construction of the BF% prediction model. The results of a multivariate linear regression analysis, which includes the HWDI and age variables, yielded a BF% for men of 34.508 − 0.159 (HWDI) + 0.161 (age) [adjusted *R*^2^ = 0.215, standard error of estimate (SEE) = 5.37%, *p* < 0.001], and, for women, 53.35 − 0.265 (HWDI) + 0.132 (Age) [adjusted *R*^2^ = 0.337, SEE = 4.39%, *p* < 0.001] (see Table 2).

## 4. Discussion

In our study, HWDI, a relatively new obesity measurement indicator, was found to have an inverse relationship with BF% in both men and women. However, Pearson's correlation coefficients were found to be low (*r* = −0.20 for men and *r* = −0.47 for women) when compared to previous studies that utilized other obesity evaluation tools with BF% [16, 18, 19]. In 1996, Gallagher et al. [20] studied the relationship between BMI and BF% and reported values of *r* = 0.58 for men and *r* = 0.72 for women. Ilman et al. [19] have reported *r* = 0.85 for men and *r* = 0.83 for women. Each study described a distinct BF% prediction model. It had been previously reported that, besides age and gender, other variables such as nationality, ethnicity, and religion can also help improve the accuracy of a BF% prediction model [16, 19–23].

A multivariate linear regression analysis showed that age and gender were statistically significant variables contributing to changes in BF%, which supports the results of previous studies [20, 24–26]. However, many of those studies used BMI as an independent variable along with the others mentioned above in constructing a BF% prediction model and found that the use of BMI introduced some limitations.

Results of this study showed that the relationship between HWDI and BF% was linear, whereas other researchers have reported different forms in the relationship between BMI and BF%, such as a curvilinear one [16, 27]. Our study concerning BF% prediction models consisting of HWDI and age grouped by gender resulted in better SEE values than that of Mott et al. where BMI was used in the prediction of BF% in four different groups of population with Asian, Black, Puerto Rican, and White ethnicity [28]. In addition, the SEE values derived from this study were similar to, yet higher than, those of some other studies [16, 29]. This may be because the *r* value between HWDI and BF% in this research was lower in comparison to the others.

Although the units in the mathematical operation in HWDI are not the same (subtracting height (in cm) from body weight (in kg)), our objective was to use HWDI as an index to predict BF% rather than using it to indicate BF% directly. We built the model to predict BF% from HWDI in relation to age and gender as an obesity screening option particularly useful in resource limited settings where gold standard body composition measurement methods such as DEXA and BIA may not be appropriate for regular use. In addition, it is a quick and simple method that does not require a great deal of training to utilize.

We compared the adjusted *R*^2^ and SEE of the BF% predictive model for both genders as a function of HWDI and age to a predictive model as a function of BMI and age. The adjusted *R*^2^ of the model based on HWDI was larger than for the model based on BMI (0.212 versus 0.151), whereas SEE was smaller in the model based on HWDI (5.760 versus 5.980). This clearly demonstrates the improvements of using HWDI over BMI.

In the study using the same set of data, Juntaping et al. [30] proposed HWDI to screen obesity for each age group by gender. In this study, the obesity was proportionately higher in women than men, which is in accordance with previous studies which showed a higher risk of obesity in women both globally and in Asia [1, 3, 31, 32]. This may be due to differences in eating and exercising behaviors from men, as well as physical attributes, hormones, and metabolism [33–35]. The sensitivity (Se) results indicated that 65% of those classified as obese by their measurements using HWDI were also classified as obese using BF%  (Se = 0.65). In addition, their specificity (Sp) results showed that 78% of those not classified as obese using HWDI were also not classified as obese using BF%  (Sp = 0.78) [30]. New-HWDI underestimated values for screening obesity status discordant with BF% in the following gender and age categories: 3.4% of men aged 18–39, 11.4% of men aged 40–59, 23.8% of men aged ≥ 60, 1% of women aged 18–39, 1.5% of women aged 40–59, and 12% of women aged ≥ 60. In comparison, disagreement between BMI and BF% values was found in 4.1% of men aged 18–39, 11.4% of men aged 40–59, 35.6% of men aged ≥ 60, 1% of women aged 18–39, 1.6% of women aged 40–59, and 11.4% of women aged ≥ 60. It is evident that New-HWDI compares well with BMI and is likely to classify obesity status with a lower proportion of underestimated values in some age groups. Indeed, New-HWDI and BMI screen obesity status were based on only height and weight using a simple calculation. However, obesity screening of the elderly may be less adequate compared to younger people because the former may have less muscle but more body fat, and they may have osteoporosis, which is often found in inhabitants of low or middle-low income countries, especially in women [36, 37].

In our study, we have extended sensitivity and specificity analyses of BMI to compare them with HWDI, while at the same time referring to BF% as the gold standard. We found that 46% of those classified as obese by their measurement of HWDI were also classified as obese by BF%  (Se = 0.46), and when considering specificity, it was discovered that 71% of those not classified as obese by their measurements of HWDI were also not considered obese by their measurements of BF%  (Sp = 0.71). This supports our findings that HWDI could be used as a way to deal with the limitations of BMI in identifying obesity in intermediate ranges.

Our predictive model derived in this study uses HWDI since it is more accurate and easier to use than BMI. This has resulted in an easier means to evaluate obesity, thus aiding the monitoring of high-risk groups in the population so as to avoid problems associated with it.

## Figures and Tables

**Table 1:** Population characteristics.

Characteristics	Men	Women	*p*
Overall, *n* (%)	999 (36.1)	1,772 (64.0)	
Median age (IOR) (years)	60 (47–68)	52 (43–60)	**<0.001**
Age groups, *n* (%)			
18–39	148 (14.8)	311 (17.6)	
40–59	351 (35.1)	959 (54.1)	
≥60	500 (50.1)	502 (28.3)	
Median weight (IOR) (kg)	63 (55–71)	56 (50–62)	**<0.001**
Median height (IOR) (cm)	165 (160–170)	155 (150–158)	**<0.001**
Median body-fat percentage (IOR)	27 (24–31)	34 (31–38)	**<0.001**
Median BMI (IOR) (kg/m^2^)	23 (21–26)	24 (21–26)	0.310
Median HWDI (IOR)	101 (95–107)	98 (92–104)	**<0.001**

*p* from Wilcoxon rank-sum test. *p* in bold corresponds to *p* < 0.05.

**Table 2:** Regression analysis for changes in BF% with HWDI, age, and gender.

Covariates	Regression coefficients	Standard error	*p*	SEE (%)	Adjusted *R*^2^
Overall			**<0.001**	4.80	0.452
Intercept	48.267	1.000			
HWDI	−0.221	0.010	**<0.001**		
Age	0.148	0.006	**<0.001**		
Gender	−6.791	0.195	**<0.001**		
Men			**<0.001**	5.37	0.215
Intercept	34.508	1.784			
HWDI	−0.159	0.017	**<0.001**		
Age	0.161	0.011	**<0.001**		
Women			**<0.001**	4.39	0.337
Intercept	53.35	1.210			
HWDI	−0.265	0.011	**<0.001**		
Age	0.132	0.008	**<0.001**		

*p* from Wald's test. *p* in bold corresponds to *p* < 0.05. SEE = standard error of estimate.
